# 多原发肺癌的诊断与治疗

**DOI:** 10.3779/j.issn.1009-3419.2015.12.09

**Published:** 2015-12-20

**Authors:** 晶晶 侯, 慧娟 王

**Affiliations:** 450008 郑州，郑州大学附属肿瘤医院呼吸内科 Department of Respiratory Medicine, the Affiliated Cancer Hospital of Zhengzhou University, Zhengzhou 450008, China

**Keywords:** 肺肿瘤, 同时性, 异时性, 诊断, 治疗, Lung neoplasms, Synchronous, Metachronous, Diagnosis, Treatment

## Abstract

多原发肺癌（multiple primary lung cancer, MPLC）是原发性肺癌的一种少见类型。随着多层螺旋计算机断层扫描（multislice spiral computed tomography, CT）及正电子发射体层显像（positron emission tomography, PET）等早期检查工具的广泛使用、原发性肺癌切除后生存率的提高，越来越多的MPLC被检出。但当前的诊断与治疗方法尚不能满足MPLC的个体化诊疗需求。目前MPLC在诊断方面主要依据肿瘤的组织学类型、遗传学特点、影像学特征、发生部位及临床表现来诊断，在治疗方面采取以手术为主的多学科综合治疗。本文总结最新的文献进展就MPLC的流行病学特征、病因、诊断、治疗及预后等方面做一综述。

## Abstract (en)

Multiple primary lung cancer (MPLC) is an uncommon entity of the primary lung cancer. With the widespread use of early detection tools such as multislice spiral computed tomography (CT), positron emission tomography (PET), and the better survival results after resection of primary lung cancer, more and more MPLC have been detected. However, the current diagnostic and therapeutic methods cannot meet the needs of MPLC individualized demand of diagnosis and treatment. At present, diagnostic criteria of MPLC are based on histological types, genetic characteristics, image features, tumor locations and clinical manifestation. On the treatment of MPLC, Surgery-based multi-discipline comprehensive treatment is regarded as the treatment of choice. In this paper, we make a review through summarizing the latest literature progress on the epidemiology, etiology, diagnostic criteria, treatment options and prognosis of MPLC.

多原发肺癌（multiple primary lung cancer, MPLC）是指同一个体，一侧或双侧肺内不同部位，同时或先后发生两个或两个以上的原发性肺癌，组织学类型可以相同或不同。MPLC中最先发生的癌称为初原发癌即初癌或首癌，其后发生的癌称为次癌或重复癌，可有多次重复癌，即多重癌。根据不同癌灶出现的时间间隔，可将MPLC分为 < 6个月的同时性MPLC（synchronous MPLC, sMPLC）和≥6个月的异时性MPLC（metachronous MPLC, mMPLC）。过去认为，MPLC是一种不常见的原发性肺癌，发病率较低，但近年来MPLC的发病率日益增高。临床上，MPLC易与肺癌的复发、转移相混淆，容易造成误诊、误治，而MPLC与肺内转移癌及复发癌的临床治疗及预后截然不同^[1-3]^，前者经手术治疗有治愈的可能，而后者基本无治愈机会，远期生存率低，因此必须明确诊断以满足MPLC的个体化治疗需求。

## 1 MPLC的流行病学特征

1924年Beyreuther^[4]^在尸检中报道了第一例双原发肺癌，此后关于MPLC的报道才开始出现，但至今仍无关于MPLC发病率的确切统计报道。

国内外报道MPLC占肺癌的比例在0.8%-14.5%之间^[3, 5-7]^。MPLC发病率变化较大可能与各地肺癌的发病率、人均寿命、肺癌早诊断、早治疗率等的不同有关。MPLC的发病率随着首癌治疗后生存期的延长而升高。首癌手术治疗后3年、5年、8年第二原发肺癌（second primary lung cancer, SPLC）的发病率分别为5%、8%、16%^[8]^。过去报道MPLC病理类型以鳞癌多见^[9]^，而目前文献报道MPLC病理类型以腺癌最多见^[1-3, 5, 10-14]^。MPLC中相同病理类型发病率高于不同病理类型，其中以腺癌-腺癌最为常见，其次为鳞癌-鳞癌^[3, 10-12]^。但mMPLC和sMPLC的流行病学特征，似乎并不太一致。

mMPLC中腺癌多于鳞癌但差异并不十分明显，次癌和首癌发生的中位时间间隔为34个月-66个月，男性多于女性，次癌中位年龄为60岁-70岁，目前吸烟者与曾经吸烟者多于从不吸烟者，多发癌灶位于双侧肺者高于同侧肺者^[7, 15, 16]^。Yang等^[15]^回顾了其中心2006年-2011年诊断的143例mMPLC患者，男性所占比例为74.1%，女性为25.9%，首癌和次癌组织学类型相同者为74.8%，首癌中腺癌比例为44.1%，鳞癌为40.6%，SPLC中腺癌占44.8%，鳞癌占37.8%，第二原发癌时患者中位年龄为60岁，SPLC与首癌的中位时间间隔为34个月，吸烟≥20包年者占48.3%，< 20包年者占51.3%。此研究并未明确说明吸烟者和不吸烟者所占的比例。

sMPLC中腺癌比例明显高于鳞癌，多发癌灶位于同侧肺者高于异侧肺者，中位年龄为65岁-68岁，女性、不吸烟者多于男性、吸烟者^[1, 7, 13, 14]^。Yu等^[13]^回顾了其中心2001年-2011年收治的97例sMPLC患者，其中男性占43.3%，女性占56.7%，中位年龄为67岁，目前和曾经吸烟者占40.2%，病理类型为腺癌-腺癌的占76.3%，鳞癌-鳞癌为5.2%，位于同侧肺者占87.6%。

## 2 MPLC的病因及发病机制

MPLC的病因与发病机制尚不清楚，根据目前的文献报道可能与以下几种原因有关：

### 2.1 人口老龄化

癌症是一个年龄相关性疾病，细胞癌变是多阶段、多基因的损伤过程，年龄越大，细胞的损伤修复功能越低，癌变的机会就越大。随着年龄的增长细胞中的DNA更容易发生点突变、缺失、扩增、易位或移位等，导致原癌基因的活化。2/3的肿瘤患者的年龄≥65岁，40%的肿瘤患者年龄 > 70岁^[17]^，54岁-64岁之间的MPLC发病率为5%-12%，而80岁以上的MPLC的发病率为12%-26%^[18]^，因而随着目前人均寿命的延长MPLC的发病率也日渐增加。

### 2.2 遗传和基因变异

MPLC与遗传及后天获得性基因缺陷有关，如细胞的微卫星不稳定、杂合性缺失、p53、HER-2、K-ras过表达等^[19, 20]^。具有肺癌家族史的肺癌患者更易发生MPLC，基于瑞典人群的一项研究表明有肺癌家族史的患者MPLC的发病率是无肺癌家族史患者的9倍，且具有明显的遗传倾向，遗传易感性可预测一小部分患者发展为MPLC^[21]^。

### 2.3 烟草暴露

吸烟是肺癌的独立危险因素，香烟导致p53突变与MPLC的发生有密切关系^[22]^。MPLC患者的香烟烟雾暴露率明显高于单原发肺癌。呼吸道长期暴露于烟草致癌物的慢性刺激下从而导致支气管粘膜的广泛异形增生，其中某些高级别异形细胞首先癌变，发展为第一原发肺癌，若致癌物长期存在可能导致其他部位相继发生癌变。Boyle等^[8]^报道首癌术前戒烟者MPLC的5年发病率为7%，首癌手术时吸烟者MPLC的5年发病率为13%，大量持续吸烟是MPLC的风险因素之一。

### 2.4 医源性因素

随着医疗水平的提高，增强CT、PET-CT等检查手段的应用使更多的肺癌患者可以早诊断、早治疗，从而提高了首癌治疗的疗效，患者生存期得以延长，加之近年来医务工作者对MPLC的认识提高使得MPLC的检出率升高。在发生首癌后进行化/放疗治疗可诱发细胞的突变，也可引起MPLC的发生。

### 2.5 区域性癌化（field cancerization）假说

该假说认为整个呼吸道都暴露在致癌因子的作用下，从而导致呼吸道多处发生癌变^[23]^，最终导致多原发癌的发生。

### 2.6 精神心理因素

患第一原发癌后焦虑、抑郁的精神心理因素对自身免疫力的影响，增加了第二癌症的发生率。

## 3 MPLC的诊断

MPLC易与原发肺癌的转移、复发及其卫星灶相混淆，加之近年来临床多倾向运用“一元论”来解释肿瘤的发生和发展，因而MPLC易被误诊为复发癌或转移癌，但它们的治疗手段以及预后又截然不同，明确诊断是实现个体化治疗的关键。

1975年由Martini和Melamed^[24]^首先提出了MPLC的诊断标准：sMPLC的诊断需要满足以下条件：肺癌发生的部位各异，彼此孤立，组织学类型不同; 组织学类型相同时，位于不同肺段，肺叶，不同侧肺，由不同的原位癌起源，肺癌共同的淋巴引流部位无癌肿，确立诊断时无肺外转移。mMPLC的诊断需满足：组织学类型不同; 组织学类型相同时，无瘤间期至少2年，或均由不同的原位癌起源，或第二原发癌位于不同肺叶、不同侧肺，肺癌共同的淋巴引流部位无癌肿，确立诊断时无肺外转移。

1995年Antakli等^[9]^将上述诊断标准修订为：组织学类型不同; 组织学类型相同时应至少满足以下任意2个条件：①各个肿瘤的解剖学位置不同; ②均有相关的癌前病变; ③无全身转移; ④无纵隔侵犯; ⑤不同肿瘤的DNA倍体不同。

2003年美国胸科医师协会（American Collge of Chest Physicians, ACCP）对Martini和Melamed提出的MPLC的诊断标准进行了修订^[25]^。修订后的MPLC诊断标准为：①组织学类型相同，解剖学位置不同，癌灶位于不同肺叶，无N2、N3转移，无全身转移; ②组织学类型相同，发病时间不同，癌灶时间间隔≥4年，无全身转移; ③组织学类型不同，或分子基因学特征不同，或分别由不同的原位癌发展而来。同时指出：组织学类型相同，伴全身转移; 位于不同肺叶有N2、N3转移; 癌灶时间间隔 < 2年的为肺转移癌。癌灶时间间隔2年-4年无全身转移的不能确定。

2007年和2013年ACCP关于MPLC的诊断仍沿用2003年标准^[26, 27]^。目前研究发现采用以上几种标准，诊断MPLC的结果并无差异^[27]^。目前临床研究中多用的仍为Martini和Melamed标准。基于以上标准，组织学类型相同时，单纯依靠肺癌病灶所在的部位、发病时间间隔、淋巴结及全身转移情况诊断MPLC仍较困难。

对于组织学类型相同的MPLC，也可利用肺癌组织学亚型区分MLPC和转移癌及复发癌，如腺癌可根据腺泡状、鳞屑样、乳头状等亚型及所占比例不同进行区分^[27]^。另外目前有许多基于分子生物学和分子遗传学水平的辅助诊断方法，这些辅助诊断方法主要有：特异的分子标记、微卫星不稳定和杂合性缺失分析、第二代基因测序、微阵列比较基因组杂交（array comparative genomic hybridization, aCGH）、免疫组化等。转移和复发性肿瘤与原发肿瘤有着相似的遗传学特征而第二原发癌与首癌的遗传学特征不同^[28]^。目前的分子诊断多基于这一理论。

目前常用的分子诊断方法为依据*EGFR*、*K-RAS*、*p53*的突变状态及ALK重排来区别MPLC和肺内转移癌。Shen等^[19]^对5例MPLC和7例肺内转移癌患者，利用6个多态微卫星标记的等位基因变异的分子分析，结果显示多态性微卫星标记在MPLC表现出不一致的趋势而在转移癌和原发癌之间表现出一致的趋势。Chen等^[20]^利用免疫组化方法检测p53、p16、p27和c-erbB2四个蛋白在肺癌病灶中的差异性表达鉴别MPLC和肺内转移，四个蛋白表达率差值总和 > 90%则诊断为MPLC，≤90%则诊断为肺内转移。Arai等^[29]^利用aCGH分析转移癌和多原发癌的基因拷贝数变化的一致率，结果发现转移癌和多原发癌的一致率分别为55.5%和19.6%具有差异，且与病理诊断的一致率高达83%。周渝斌等^[30]^应用微量全血单细胞凝胶电泳技术检测MPLC、原发肺癌、转移癌患者外周血单个有核细胞DNA损伤率和损伤程度结果显示转移癌组单个有核细胞DNA损伤率和损伤程度高于MPLC组，而MPLC组又高于原发肺癌组。

然而，一些研究^[31-33]^表明，在肺原发癌与转移癌之间也存在分子异质性。例如，方勤等^[31]^研究报道，非小细胞肺癌（non-small cell lung cancer, NSCLC）原发灶与转移灶的*EGFR*基因表达存在不一致性。Chang等^[33]^报道NSCLC原发灶和转移灶的EGFR和p53突变状态存在差异。ACCP^[27]^认为目前基于分子生物学方面的诊断只能做为参考。

MPLC的影像学诊断多采用高分辨率螺旋CT平扫加增强通过观察多发癌病灶的位置、大小、形态、胸膜牵拉、与周围组织的关系、结节内部性质的不同来鉴别诊断。原发性肺癌结节内部密度多不均匀，有空泡征、空洞、多结节聚集、空气支气管征; 形态多为分叶形或不规则形，其中深分叶的诊断价值最高; 周围多有毛刺征、棘突征、血管集束征、胸膜凹陷征^[34, 35]^。另外，肺癌的CT征象与肿瘤的分化程度存在相关性，结节实性成分越多提示分化越差，毛刺征与胸膜牵拉征在低分化腺癌中最常见，因此同一患者多原发癌灶影像学表现有差异，可提示为不同起源的多原发癌^[35]^。对于初诊不能定性的病灶应进行严格的影像随访，若随访过程中出现病灶增大，或病灶大小无变化但密度增高，内部出现实性成分或实性成分增多，则提示恶性机率大^[10]^。

ACCP^[27]^认为有经验的放射科、肺内科、胸外科、病理科医师组成的多学科团队对于MPLC的诊断最为重要，多学科团队可通过肿瘤的组织学类型、分子遗传学特性、影像学特征、肿瘤的发生部位及临床表现来诊断。

## 4 MPLC的治疗

目前对于MPLC的治疗仍无统一的认识。但国内外文献对于MPLC的治疗原则是一致的，均认为只要无绝对禁忌症，应行积极的以手术为主的局部治疗，结合放化疗辅助治疗的多学科综合治疗模式^[7, 14-16, 36]^。

Pairolero等^[37]^报道mMPLC的2年生存率为52%，而转移癌和复发癌分别为23%和9%。这些数据表明MPLC是潜在可治愈性疾病，而转移性及复发性肺癌的预后通常较差^[26]^。转移性或复发性肺癌通常不能手术治疗，多采用化疗、放疗、靶向治疗等姑息性治疗，远期生存率低。然而如果肺内多发结节均为原发，有足够肺储备功能，分期为早期可以手术治疗的患者，积极的手术治疗可使患者生存明显获益，且相同分期的sMPLC与原发性肺癌的预后相似^[13]^。Ⅰ期sMPLC的5年生存率可高达75.8%^[1]^。MPLC手术原则为“两个最大限度”，即最大限度地保存正常肺组织和最大限度地切除肿瘤。另外，手术前后的辅助治疗是有利的预后因素^[38]^。

sMPLC手术切除原则为：如果病灶位于同侧肺内则尽可能1次手术同时切除; 位于双侧肺者先切除分期较晚一侧的病灶，待患者完全恢复并有足够的肺功能储备后择期手术切除对侧病灶，两次手术的间隔时间通常为1个月左右，先切除中央型肺癌或进展较快的病灶，后切除周围型肺癌; 先切除体积大者，后切除体积小者; 先切除临床诊断有纵隔或肺门淋巴结转移的肿瘤，后切除无淋巴结转移的肿瘤^[14, 39]^。但无论何种手术，系统的淋巴结清扫都是必要的^[14]^。文献^[7, 14]^报道无论病变位于单侧或双侧，如果肺功能储备良好淋巴结阴性的患者均应尽可能采取肺叶切除，如果病例无法承受肺叶切除术，可选择亚肺叶切除，另外应尽量避免全肺切除^[2, 7]^。一些研究^[3]^结果表明虽然亚肺叶切除有较高的复发率但从长期生存来看肺叶切除对比亚肺叶切除并无显著差异。然而另一些研究^[7, 14]^则证明肺叶切除的5年生存率优于亚肺叶切除。双侧sMPLC分期手术相对安全，但是有可能因为二次手术时间间隔长而导致肿瘤播散或转移的风险^[3]^。

mMPLC的治疗受患者的肺功能、年龄、相关的合并症、重复癌的临床分期、首癌的治疗方式等多种因素影响^[7]^。若患者无手术禁忌症，应积极选用手术治疗^[12, 16]^。Hamaji等^[16]^报道SPLC术后5年生存率和首癌术后5年生存率基本相同，评估SPLC能否进行手术的评估方法同首癌的评估方法相同，分期为早期有足够生理储备的mMPLC可行手术治疗。手术方式原则上与首癌手术方式相同即肺叶切除术^[15]^。肺叶切除是早期肺癌的标准治疗，然而重复癌如果和首癌位于同侧肺内或患者心肺功能差，此时可选择亚肺叶切除^[7, 12, 15, 16]^，另外，全肺切除是独立的不良预后因素，应尽量避免全肺切除^[7, 15]^。

有大量吸烟史、肺功能储备差及有其他医疗合并症不能行手术治疗的早期中心性肺癌患者，可行光动力治疗^[30-42]^。Sokolov等^[43]^报道光动力治疗早期 < 1 cm中心性肺癌的完全缓解率可达100%。另外，一般状况差、合并症状多不能行手术治疗的早期MPLC患者也可以选择立体定向放射治疗（stereotactic body radiation therapy, SBRT）^[44]^。SBRT有良好的长期肿瘤控制率和生存率及较小的毒性，Chang等^[45]^报道应用SBRT治疗早期MPLC的2年和4年生存率分别为73.2%和47.5%，3级放射性肺炎发生率为3%。

晚期MPLC患者可行放疗、化疗、靶向治疗、介入治疗、免疫治疗、最佳支持治疗等姑息性治疗。

## 5 MPLC的预后

文献报道^[2, 5, 13-16, 37, 45]^行手术治疗的MPLC患者的生存期明显优于未行手术治疗的患者，手术者中位生存期为32个月-49个月，sMPLC的5年生存率为35%-87%，mMPLC的5年生存率为37.0%-60.8%，均明显优于Ⅳ期肺癌患者5年生存率。淋巴结受累是关联最强的预后因素^[3]^，淋巴结无受累的MPLC患者术后5年生存率可达45%-87%^[2, 3, 45]^。第1秒用力呼气量、非最佳的手术方式、全肺切除术、肿瘤直径 > 2 cm、病灶位于双侧肺内、p53和HER-2过表达是不良预后的独立预测因子，而女性、年轻、疾病无症状、分期为Ⅰ期淋巴结无受累、肿瘤直径≤2 cm是有利的预后因素^[2, 3, 13-16, 36]^。肿瘤的数目、肿瘤病理类型是否相同、亚肺叶切除对总生存无明显影响^[3, 13, 15, 16, 46]^，戒烟者和从不吸烟者总生存明显优于当前吸烟者^[8]^。

## 6 小结

随着肺癌高危患者筛查意识的提高，医疗技术的发展及医务工作者对MPLC的认识增加，MPLC发病率明显升高，而目前仍缺乏针对MPLC诊断及治疗的最佳标准。对于肺部多发的癌病灶，目前多借助病理类型、分子生物学及分子遗传学特征、影像学表现等诊断，我们期待今后可以利用简便有效的方法来诊断MPLC。目前治疗方面可手术治疗的MPLC多采用手术治疗。不能手术的早期MPLC可采用光动力、SBRT等方法治疗，晚期患者多采用放化疗、靶向治疗、免疫治疗相结合的综合治疗模式。我们相信不久的将来会有更多关于MPLC诊断与治疗的研究进展及更多新的诊断与治疗方案的出现。
